# Improved Real-Time Detection Transformer with Low-Frequency Feature Integrator and Token Statistics Self-Attention for Automated Grading of *Stropharia rugoso-annulata* Mushroom

**DOI:** 10.3390/foods14203581

**Published:** 2025-10-21

**Authors:** Yu-Hang He, Shi-Yun Duan, Wen-Hao Su

**Affiliations:** 1 College of Engineering, China Agricultural University, 17 Qinghua East Road, Haidian, Beijing 100083, China; 256092303@mail.sit.edu.cn (Y.-H.H.); 2022307140305@cau.edu.cn (S.-Y.D.); 2 Faculty of Intelligence Technology, Shanghai Institute of Technology, 500 Haiquan Road, Fengxian District, Shanghai 201418, China

**Keywords:** *Stropharia rugoso-annulata* mushroom, RT-DETR, low-frequency feature integrator, token statistics self-attention, real-time detection

## Abstract

Manual grading of *Stropharia rugoso-annulata* mushroom is plagued by inefficiency and subjectivity, while existing detection models face inherent trade-offs between accuracy, real-time performance, and deployability on resource-constrained edge devices. To address these challenges, this study presents an Improved Real-Time Detection Transformer (RT-DETR) tailored for automated grading of *Stropharia rugoso-annulata*. Two innovative modules underpin the model: (1) the low-frequency feature integrator (LFFI), which leverages wavelet decomposition to preserve critical low-frequency global structural information, thereby enhancing the capture of large mushroom morphology; (2) the Token Statistics Self-Attention (TSSA) mechanism, which replaces traditional self-attention with second-moment statistical computations. This reduces complexity from $O(n^{2})$ to O(n) and inherently generates interpretable attention patterns, augmenting model explainability. Experimental results demonstrate that the improved model achieves 95.2% mAP@0.5:0.95 at 262 FPS, with a substantial reduction in computational overhead compared to the original RT-DETR. It outperforms APHS-YOLO in both accuracy and efficiency, eliminates the need for non-maximum suppression (NMS) post-processing, and balances global structural awareness with local detail sensitivity. These attributes render it highly suitable for industrial edge deployment. This work offers an efficient framework for the automated grading of large-target crop detection.

## 1. Introduction

*Stropharia rugoso-annulata*, also known as the wine cap mushroom or garden giant mushroom, is a highly valuable edible fungus with significant economic value in both domestic and international markets [1]. As one of the recommended edible mushrooms by the Food and Agriculture Organization of the United Nations, it is characterized by high protein content, abundant bioactive compounds, palatable flavor, and pleasant aroma. In industrialized production, fresh *Stropharia rugoso-annulata* products are sold by grade, with prices varying significantly across grades. Currently, grading primarily relies on manual sorting, a subjective and labor-intensive process that accounts for a large proportion of labor costs in industrialized production. Due to the lack of consistent objective criteria, manual sorting suffers from heavy workloads, low efficiency, and inconsistent standards—issues that severely hinder the development of post-harvest processing for this mushroom [2].

Automatic detection and grading of *Stropharia rugoso-annulata* is therefore crucial for improving sorting efficiency and reducing labor costs in industrial settings. However, real-time sorting of unshelled mushrooms remains challenging due to difficulties in accurately identifying, locating, and classifying large quantities of specimens [3]. Further complicating the challenge, these models must be deployable on resource-constrained edge devices, creating a fundamental tension between maintaining high accuracy and achieving lightweight architectural design.

Automated grading of agricultural products has attracted growing attention, yet the grading of large-sized objects such as *Stropharia rugoso-annulata* mushrooms remains particularly challenging. Existing detectors often struggle to maintain both global structural awareness and sensitivity to local details, leading to poor recognition of large targets with subtle surface variations. Furthermore, many transformer-based models achieve strong accuracy but incur high computational complexity, making them difficult to deploy on resource-constrained edge devices commonly used in agricultural environments.This gap—balancing global structural modeling with local feature sensitivity under real-time and edge deployment constraints—remains underexplored. Addressing it is crucial for ensuring that automated grading systems can be both accurate and practical in real-world post-harvest operations. In this work, we aim to close this gap by enhancing RT-DETR with lightweight feature fusion and efficient attention mechanisms, enabling reliable real-time grading of large agricultural mushrooms.

In contemporary object detection research, mainstream approaches primarily fall into two categories: (1) the YOLO (You Only Look Once) family, which prioritizes real-time inference through streamlined convolutional architectures, and (2) the DETR (Detection Transformer) family, which introduces end-to-end detection frameworks based on attention mechanisms, offering improved performance in complex scenarios.

The You Only Look Once (YOLO) series [4] has emerged as a dominant paradigm for real-time object detection, striking an effective balance between computational efficiency and detection accuracy [5]. Within agricultural computer vision applications, YOLO-based architectures have shown particular promise for *Stropharia rugoso-annulata* detection. A notable example is APHS-YOLO, which integrates YOLOv8n with AKConv, CSPPC, and HSFPN modules to create a lightweight model for identifying *Stropharia rugoso-annulata* of different grades and seasons [6]. And Lv et al. employed the YOLOv8-seg instance segmentation algorithm to precisely delineate the cap and stalk regions of *Stropharia rugoso-annulata* mushrooms, enabling quantitative analysis of morphological features for automated quality grading [7]. However, despite these advancements, YOLO-series detectors remain fundamentally constrained by their dependence on non-maximum suppression (NMS) post-processing. This computationally intensive operation not only introduces latency but also presents significant deployment challenges on edge devices with strict resource constraints.

Although DETR significantly simplifies the detection pipeline by eliminating NMS, its adoption of more complex transformer architectures results in increased computational costs [8]. This inherent complexity makes direct deployment on edge devices particularly challenging. Nevertheless, DETR’s superior feature extraction capabilities enable it to outperform YOLO-series models in accuracy, garnering substantial research attention [9].

The Real-Time Detection Transformer (RT-DETR), the first real-time variant in the DETR series, shows promising potential for practical deployment in agricultural edge computing scenarios. As a notable advancement in object detection, RT-DETR integrates the efficiency of CNN-based models with the global context comprehension capabilities of transformers. Its encoder employs an efficient hybrid architecture to handle multi-scale features by decoupling internal scale interactions and cross-scale fusion, reducing computational costs for real-time detection. Its decoder, a multi-layer transformer, allows flexible selection of decoder layers during inference to adjust speed without retraining. Recent studies across domains confirm RT-DETR’s effectiveness but reveal key limitations: He et al. optimized it for diabetic retinopathy detection, achieving 0.90 precision, 0.85 recall, and 0.88 mAP50 on EyePACS (outperforming YOLOv5 on small targets) but struggled with uneven data and complex backgrounds [10]; Yao et al. enhanced it for maize leaf disease detection via DAttention, SCConv, and lightweight QARepVGG, boosting mAP50 by 7.3% to 92.0% with 18.9M fewer parameters, yet misjudging targets amid soil and weeds [11]; Liu et al.’s WRRT-DETR improved weather robustness via GLCE, FSAE, and ACFM, achieving 82.3% mAP50 on AWOD (20.2M parameters, 66.4 FPS) but missing small targets in low light and lagging behind YOLO models in speed [12]; Sun et al.’s RTDETR-MARD used feature aggregation and WIoU loss for aquatic waste detection, hitting 86.6% mAP50 on FloW but lacking edge deployment and risking false detections in complex environments [13]. These studies highlight RT-DETR’s limitations such as poor performance on large objects, high computational complexity, and challenges in edge deployment—gaps that motivate our targeted improvements for *Stropharia rugoso-annulata* detection.

*Stropharia rugoso-annulata* detection presents unique challenges, particularly due to the mushroom’s large size, which can dominate images and requires balancing global structural capture with fine-detail extraction. To address this, we introduce a low-frequency feature integrator (LFFI) module in the backbone’s convolutional downsampling operations. The LFFI module uses wavelet decomposition to extract and preserve low-frequency information—critical for capturing the global structure of large objects—thereby enhancing the model’s ability to accurately detect and localize large mushrooms while retaining sensitivity to details essential for grading.

A second challenge lies in the quadratic computational complexity of traditional transformer models (including RT-DETR), which stems from pairwise similarity calculations between tokens and limits real-time performance on resource-constrained devices [14]. Recent advances in linear attention have sought to mitigate the quadratic complexity of softmax attention. Linformer [15] compresses sequence length via low-rank projections, Performer [16] employs random feature approximations for kernelized attention, and Nyströmformer [17] leverages landmark-based approximations. Katharopoulos et al. [18] proposed Linear Transformers that reformulate attention as a kernel feature map. These approaches reduce time and memory complexity while retaining reasonable accuracy. Building on this line of work, we replace RT-DETR’s standard self-attention with a Token Statistics Self-Attention (TSSA) mechanism [19], which reduces complexity to linear levels in both computation and memory. By introducing TSSA, our model achieves comparable accuracy to traditional transformer-based models while operating much more efficiently, making it suitable for real-time applications on a wider range of devices.

In summary, this paper presents an Improved RT-DETR model for real-time *Stropharia rugoso-annulata* detection and grading, with three key innovations: (1) elimination of NMS post-processing inherent in YOLO-based models; (2) the LFFI module for enhanced large-object detection; and (3) the TSSA mechanism for efficient attention. Experimental results demonstrate significant improvements in accuracy, speed, and efficiency compared to both YOLO-based models and the original RT-DETR, achieving 95.2% mAP, and over 200 FPS—performance characteristics well-suited for industrial and agricultural deployment.

## 2. Materials and Methods

### 2.1. Dataset Source

The *Stropharia rugoso-annulata* dataset utilized in this study is derived from the grading detection dataset constructed by us. It includes samples across different seasons (spring and autumn) and grades (first, second, and third), thereby effectively supporting real-time mushroom grading tasks.

Raw images in the dataset were collected from the experimental base of the Research Institute in Pinggu District, Beijing, using an MV-UBD130C industrial camera (MindVision, Shenzhen, China). The camera parameters are as follows: spatial resolution of 1280×960, frame rate of 35 FPS, a 4-megapixel lens with a 6 mm focal length, and a fixed shooting distance of 20 cm. Image acquisition was conducted daily between 12:00 and 16:00 to ensure consistent lighting conditions. The original dataset contains 3032 images, which were expanded to 9170 images via image augmentation techniques (e.g., rotation, noise addition, and brightness adjustment). To increase sample diversity, we applied the following data augmentation operations: random rotation ($\pm 15^{\circ }$), random scaling (0.9–1.1), brightness adjustment (±20%), Gaussian noise (σ∈[0,0.01]), and horizontal flipping (probability 0.5). To prevent data leakage, all augmented variants generated from a given original image were restricted to remain within the same split (train, validation, or test). Thus, augmented samples cannot cross splits. These augmentation methods aim to enhance the model’s generalization ability and robustness in complex environments.

The dataset was split into training, validation, and test sets at an 8:1:1 ratio, yielding 7336 training images, 917 validation images, and 917 test images. The collected dataset contains three grading categories of *Stropharia rugoso-annulata*. Table 1 reports the per-grade sample counts in the training, validation, and test sets, together with the class-imbalance ratios.

Annotation was performed using the Labelme 4.5.13 tool, with bounding boxes marking *Stropharia rugoso-annulata* of different grades. Each annotated sample was directly assigned one of three labels: Autumn_First_Grade, Autumn_Second_Grade, or Autumn_Third_Grade, according to the grading criteria. The annotation standards strictly adhere to the grading criteria defined by Liu et al. [6], which are based on the ratio of cap diameter to height (RDHP) and the ratio of stalk length to diameter (RLDS) (see Table 2 for details).

### 2.2. The Network Structure of the Improved RT-DETR

**Figure 1:**
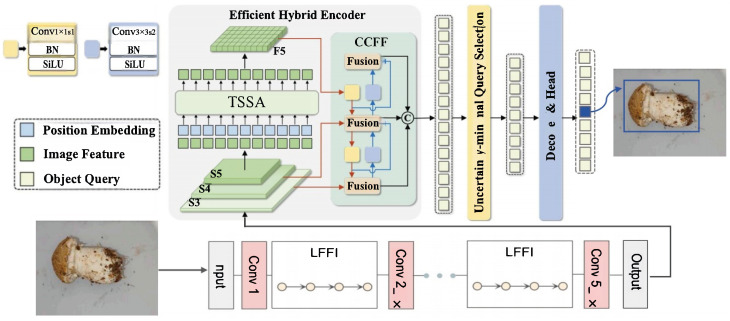
The network structure of the Improved RT-DETR.

To overcome the dual challenges of large-scale object detection and computational efficiency, we propose an Improved RT-DETR architecture specifically optimized for real-time *Stropharia rugoso-annulata* detection and quality grading. Our improved framework introduces two key innovations: (1) a low-frequency feature integrator (LFFI) for effective large-mushroom feature extraction, and (2) a Token Statistics Self-Attention (TSSA) mechanism for linear-complexity processing, enabling practical deployment on edge devices while maintaining grading accuracy. The frame diagram of the model is shown in Figure 1. Backbone selects ResNet-18 [20].

#### 2.2.1. LFFI (Low-Frequency Feature Integrator) Module

In conventional CNN-based backbones, downsampling operations tend to lose essential global shape information [21]. To overcome this limitation, we introduce a LFFI (low-frequency feature integrator) module. The LFFI module is specifically designed to address the challenge of capturing global structural information for large *Stropharia rugoso-annulata* specimens, which often occupy large areas in input images. For large targets, accurate detection and grading rely heavily on low-frequency information, such as overall shape, contour integrity, and global morphological features. However, traditional CNN-Pool architectures in backbone networks tend to focus on extracting high-frequency details (e.g., edge textures, local protrusions) through repeated convolution and pooling operations, and these operations inherently suppress or lose low-frequency components critical for representing the global structure of large objects [22]. This limitation degrades the detection and grading performance for large *Stropharia rugoso-annulata*, making it necessary to integrate a dedicated module for low-frequency feature preservation and enhancement. Given the unique advantages of wavelet transform in low-frequency information extraction and multi-scale analysis [23], it is selected as the core technology for the LFFI module, with details as follows:

**Wavelet Transform**

Wavelet transform encompasses two complementary frameworks: Continuous Wavelet Transform (CWT) for flexible time-frequency analysis, and Discrete Wavelet Transform (DWT) for efficient computation via multi-resolution analysis (MRA) [24]. For a signal f(t), the CWT decomposes it by correlating with continuously scaled and translated wavelet bases [25]. Mathematically,

(1) $$W_{f}\left(a,\tau \right)=\frac{1}{\sqrt{\left|a\right|}}\int_{-\infty }^{\infty }f\left(t\right)\psi *\left(\frac{t-\tau }{a}\right)dt,$$

where *a* is the scale parameter (controlling frequency resolution by adjusting the stretch of the wavelet basis), τ is the translation parameter (controlling spatial localization by shifting the wavelet basis), and $\psi^{*}$ denotes the complex conjugate of the mother wavelet ψ(t), where ψ(t) is a finite-energy function with zero mean, serving as the basic unit for capturing frequency-domain features.

While CWT offers flexibility, its continuous parameters (a,τ) make it computationally intensive. To enable efficient implementation, MRA constructs orthogonal wavelet bases using wavelet functions (for high-frequency details) and scale functions (for low-frequency approximations) [26].

The scale function ϕ(t), associated with the mother wavelet, satisfies the following two-scale equation:

(2) $$\phi \left(t\right)=\sum_{k}h\left(k\right)\sqrt{2}\phi \left(2t-k\right)$$

where h(k) is the scaling filter. (2t−k) enforces dyadic scaling (scale factor 1/2) and shifting (by k/2), and $\sqrt{2}$ ensures energy normalization under scaling.

Within MRA, the mother wavelet ψ(t) is designed to span the high-frequency space complementary to the scaling function’s low-frequency space [27]. It follows a parallel two-scale relation with a discrete high-pass filter g(k):

(3) $$\psi \left(t\right)=\sum_{k}g\left(k\right)\sqrt{2}\phi \left(2t-k\right)$$

with $g\left(k\right)={\left(-1\right)}^{k}h\left(1-k\right)$ ensuring the wavelet basis is biorthogonal. The scale function is responsible for approximating the low-frequency components of the signal, while the wavelet function captures high-frequency details.

Compared to traditional Fourier transform and short-time Fourier transform (STFT), wavelet transform offers distinct advantages: First, Fourier transform provides global frequency information but loses spatial localization, making it unsuitable for analyzing non-stationary signals [28]. Second, STFT uses a fixed time window to balance time and frequency resolution, but its resolution is constant across all frequencies, limiting adaptability to signals with varying frequency components [29]. In contrast, wavelet transform achieves multi-resolution analysis through the variable scale parameter *a*: it provides higher frequency resolution in low-frequency regions (large scales) and higher spatial resolution in high-frequency regions (small scales), thus balancing global structure and local detail representation [30].

**2D Image Wavelet Decomposition**

For 2D images, wavelet decomposition is extended via tensor products of 1D wavelet and scale functions, resulting in a multi-level hierarchical decomposition. Each decomposition step splits the image into four sub-bands:

- **Low frequency (LL)**: Approximates the original image, retaining global structural information.
- **Horizontal high frequency (LH)**: Captures horizontal edge details.
- **Vertical high frequency (HL)**: Captures vertical edge details.
- **Diagonal high frequency (HH)**: Captures diagonal edge details.

**Figure 2:**
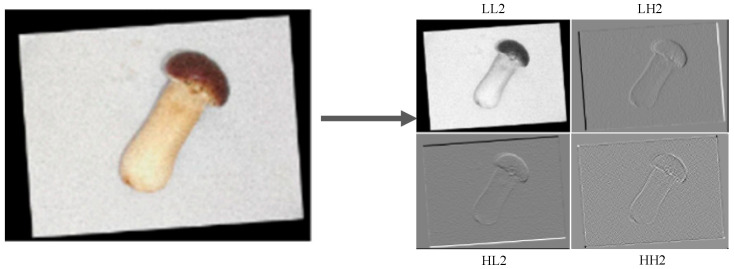
The result of an image after two rounds of Haar wavelet decomposition.

Figure 2 shows the result of an image after two rounds of Haar wavelet decomposition. This decomposition allows selective retention of low-frequency information (LL) while discarding redundant high-frequency components, ensuring efficient preservation of global structural features for large targets [31].

**Structure and Workflow of the LFFI Module**

**Figure 3:**
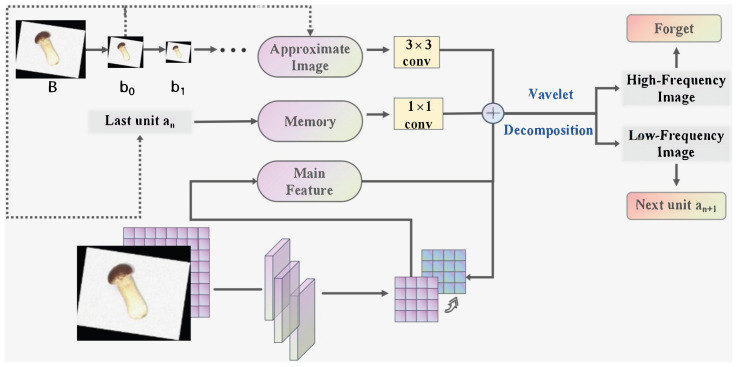
The workflow of each LFFI unit.

The LFFI module consists of a series of cascaded units, each responsible for integrating low-frequency information from wavelet decomposition into the backbone’s convolutional feature stream. The workflow of each unit is as follows (Figure 3).

1. Input Components
  Each unit *n* receives three inputs:
  **Memory feature $a_{n}$**: Output of the previous unit, representing accumulated low-frequency information from prior decompositions.
  **Approximate image $b_{n}$**: Low-frequency (LL) sub-band from the *n*-th wavelet decomposition of the original image, containing global structural information at scale *n*. For the initial unit (n=0), the memory feature is initialized as $a_{0}=b_{0}$.
  **Main feature map**: Output of the *n*-th convolutional downsampling layer in the backbone, rich in high-frequency details but lacking low-frequency context.
2. Low-Frequency Fusion
  The approximate image $b_{n}$ and memory feature $a_{n}$ are first processed via convolutional layers to adjust their channel dimensions to match the main feature map (without altering spatial resolution). These adjusted features are then added element-wise to the main feature map, resulting in an updated main feature map that integrates low-frequency global structure with high-frequency local details.
3. Memory Update
  The fused feature map (after step 2) undergoes another wavelet decomposition, from which only the low-frequency (LL) sub-band is retained, while the other three sub-bands are forgotten. This sub-band serves as the memory feature $a_{n+1}$ and is passed to the next unit, ensuring cumulative preservation of low-frequency information across multiple scales.

By iteratively integrating low-frequency approximations from wavelet decomposition into the backbone’s feature stream, the LFFI module enhances the model’s ability to capture the global structure of large *Stropharia rugoso-annulata* while retaining high-frequency details necessary for grading, thus addressing the limitation of traditional CNNs in low-frequency feature preservation.

#### 2.2.2. The Token Statistics Self-Attention (TSSA)

**Figure 4:**
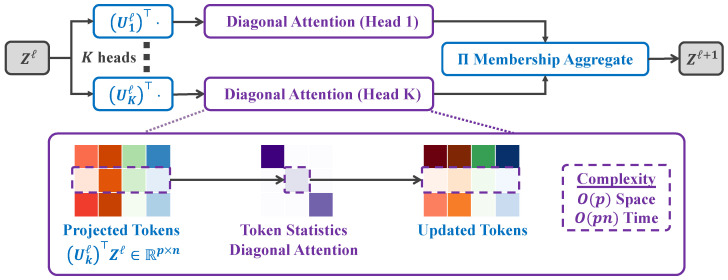
One layer *l* of the proposed Token Statistics Self-Attention (TSSA) mechanism.

The self-attention mechanism of the traditional transformer generates an attention weight matrix by calculating the similarity between all token pairs, and then performs a weighted summation over the input tokens. This results in both time and memory complexity of $O(n^{2})$ (where *n* is the number of tokens), making it difficult to handle long-sequence tasks. The Token Statistics Self-Attention (TSSA) mechanism achieves linear computational complexity O(n) through an innovative reformulation of attention computation based on the variational form of Maximal Coding Rate Reduction (${\mathrm{MCR}}^{2}$) [19]. Unlike traditional self-attention, TSSA avoids pairwise similarity calculations and realizes the attention mechanism solely through second-moment statistics of token features.The structure diagram of TSSA is shown in Figure 4.

TSSA’s innovation is rooted in the design of ${\mathrm{MCR}}^{2}$’s objective function, which provides the theoretical basis for replacing similarity-based computations. The objective function of ${\mathrm{MCR}}^{2}$ is defined as follows:

(4) $$\Delta R\left(Z,\Pi \right)≐R\left(Z\right)-R_{c}\left(Z,\Pi \right)$$

(5) $$R\left(Z\right)=\frac{1}{2}\log\det{\left(I+\frac{d}{\epsilon^{2}}\frac{1}{n}ZZ^{⊤}\right)}_{.}$$

(6) $$R_{c}\left(Z,\Pi \right)=\frac{1}{2}\sum_{k=1}^{K}\frac{n_{k}}{n}\log\det\left(I+\frac{d}{\epsilon^{2}}\frac{1}{n_{k}}Z\mathrm{Diag}\left(\pi_{k}\right)Z^{⊤}\right).$$

R(Z) is the expansion term, measuring the distribution volume of all features and encouraging feature diversity; $R_{c}\left(Z,\Pi \right)$ is the compression term, measuring the volume of features within each group and encouraging intra-group aggregation. $Z\in R^{d\times n}$ denotes the token feature matrix, and $\Pi \in R^{n\times K}$ represents the grouping probability matrix. Rows of Π denote each token’s membership probabilities across *K* groups (subspaces), and columns correspond to individual groups. $\pi_{k}$ is the probability that a token belongs to group *k*, and $n_{k}=\langle \pi_{k},1\rangle$ (where 1 is an all-ones vector with the same dimension as $\pi_{k}$).

To reduce the computational complexity of $R_{c}\left(Z,\Pi \right)$, TSSA proposes the variational upper bound $R_{c,f}^{\mathrm{var}}$, which transforms high-dimensional matrix operations into diagonal element calculations via the orthogonal matrix $U_{k}$:

(7) $$R_{c,f}^{\mathrm{var}}\left(Z,\Pi \right)≐\frac{1}{2}\sum_{k=1}^{K}\frac{n_{k}}{n}\sum_{i=1}^{d}f\left(\frac{1}{n_{k}}{\left(U_{k}^{⊤}Z\mathrm{Diag}\left(\pi_{k}\right)Z^{⊤}U_{k}\right)}_{ii}\right).$$

Here, $U_{k}\in O\left(d\right)$ is an orthogonal matrix, and *f* is a concave function. By optimizing $R_{c,f}^{\mathrm{var}}$ through gradient descent, the token update formula of TSSA is derived:

(8) $$z_{j}^{+}=z_{j}-\frac{\tau }{n}\sum_{k=1}^{K}\Pi_{jk}U_{k}D\left(Z,\pi_{k}|U_{k}\right)U_{k}^{⊤}z_{j}$$

In this formula, $D_{k}$ is a diagonal matrix whose elements are determined by the second-order moment of the projected features; τ is the learning rate; and $\Pi_{jk}$ (j=1,…,n;k=1,…,K) represents the probability that the *j*-th token belongs to the *k*-th group.

In our Improved RT-DETR, we replace the AIFI module (base on the traditional self-attention mechanism) with TSSA for intra-scale feature interaction at S5. The specific process is as follows: first, token features are projected into a low-dimensional subspace via the learnable group orthogonal matrix $U_{k}$; second, the second-order moment of each group of projected features is calculated to generate the diagonal matrix $D_{k}$; finally, the projected features are weighted and updated based on $D_{k}$, and the original features are fused via residual connection.

In summary, our proposed architecture achieves optimal performance through two synergistic innovations: (1) the LFFI module, which significantly improves global structure perception through wavelet-based low-frequency feature integration, and (2) the TSSA mechanism, which maintains computational efficiency. This combined approach results in a highly accurate yet lightweight framework that meets the stringent requirements of real-time mushroom quality grading in agricultural applications.

## 3. Experiment

### 3.1. Experiment Environment Setting

This study utilized the PyTorch 1.11.0. All experiments were conducted on an Ubuntu 20.04 operating system with CUDA 11.3, and model training and validation were performed using NVIDIA RTX 3090 24G GPUs. The specific experimental hyperparameters are presented in Table 3.

### 3.2. Evaluation Metrics

To ensure the rigor of this study and boost the credibility and value of data comparisons, the evaluation of our Improved RT-DETR leverages well-recognized assessment metrics. These include precision (P), recall (R), mean average precision (mAP), and frames per second (FPS). These metrics have seen extensive use in benchmark studies such as PASCAL VOC [32] and MS COCO [33], which validates the validity and generalizability of our chosen evaluation metrics. Moreover, to more effectively reflect the lightweight performance of the model, we have integrated metrics such as the model’s floating-point operations (FLOPs) and peak memory usage (PMU). FLOPs reflect the computational cost per image during inference, while PMU quantifies the highest memory consumption during the model’s runtime. The relevant formulas are presented below:

Precision (P) quantifies the proportion of correctly predicted positive samples among all samples predicted as positive. Mathematically, it is defined by Equation (9):

(9) $$P=\frac{\mathrm{TP}}{\mathrm{TP}+\mathrm{FP}}.$$

Recall (R) shows the number of truly positive samples that are correctly predicted as positive, as given in Equation (10):

(10) $$R=\frac{\mathrm{TP}}{\mathrm{TP}+\mathrm{FN}}.$$

Mean average precision (mAP) is used to compute the average precision (AP) across multiple categories. First, for each category *k*, the average precision ${\mathrm{AP}}_{k}$ is calculated as shown in Equation (11), which involves integrating the precision–recall curve. Then, mAP is the average of these category-level APs, as defined in Equation (12).

(11) $${\mathrm{AP}}_{k}=\int_{0}^{1}p\left(r\right)dr.$$

Here, p(r) is the precision function with respect to recall *r*.

(12) $$\mathrm{mAP}=\frac{1}{n}\sum_{k=1}^{n}{\mathrm{AP}}_{k},$$

where *n* is the total number of categories. Definitions of the four evaluation outcomes are as follows:

**True Positive (TP)**: The model predicts a positive class, and the ground-truth label is also positive, with the prediction being correct (i.e., the predicted bounding box/region matches the target).

**False Negative (FN)**: The model predicts a negative class, but the ground-truth label is positive, resulting in an incorrect prediction (i.e., a target is missed).

**False Positive (FP)**: The model predicts a positive class, but the ground-truth label is negative, resulting in an incorrect prediction (i.e., a non-target is falsely detected as a target).

**True Negative (TN)**: The model predicts a negative class, and the ground-truth label is also negative, with the prediction being correct (i.e., no false detection in a non-target region).

### 3.3. Ablation Experiments

To rigorously assess the individual and combined contributions of the proposed LFFI and TSSA modules, a series of ablation experiments were conducted using the RT-DETR baseline. The results, systematically summarized in Table 4, reveal distinct performance characteristics associated with each module, as well as their synergistic effects.

#### 3.3.1. Impact of the LFFI Module

Incorporating the LFFI module alone led to a significant improvement in detection accuracy: the mean average precision (mAP@0.5:0.95) increased from 0.917 to 0.947, representing a 3.0% gain. Notably, this enhancement was achieved with only marginal increases in model parameters and computational overhead, highlighting the module’s effectiveness in preserving low-frequency structural information—a critical factor for accurately capturing the morphology of large targets. By leveraging wavelet-based fusion of global approximations (LL sub-bands), LFFI directly mitigates the limitation of conventional CNNs in modeling holistic mushroom contours, thereby refining the precision of grading tasks.

#### 3.3.2. Impact of the TSSA Module

Replacing the original intra-scale feature interaction module (AIFI) with TSSA yielded substantial efficiency gains. When deployed independently, TSSA reduced FLOPs by 31.9% and parameters by 11.9%, while accelerating inference speed to 267 FPS—a 23.0% improvement over the baseline. Importantly, this efficiency boost did not compromise accuracy: precision and mAP@0.5:0.95 increased to 0.949 and 0.924, respectively. These results validate TSSA’s ability to maintain discriminative power through its linear-complexity statistical attention mechanism.

#### 3.3.3. Synergistic Effects of LFFI and TSSA

The combined integration of both modules achieved an optimal balance between performance metrics. The full model (LFFI + TSSA) reached a mAP@0.5:0.95 of 0.952, exceeding the baseline by 3.8%, while operating at 262 FPS—20.7% faster than the original architecture. Furthermore, this configuration reduced FLOPs by 27.3% and parameters by 12.9%. These results demonstrate that LFFI’s focus on global structural awareness and TSSA’s sensitivity to local details are complementary rather than conflicting. This co-design effectively resolves the longstanding trade-off between accuracy and efficiency in agricultural vision systems.

### 3.4. Comparison Experiments

To validate the industrial applicability of the proposed model, comparative evaluations were conducted against two benchmarks: APHS-YOLO and the original RT-DETR. As summarized in Table 5, the Improved RT-DETR demonstrates significant advantages across key metrics, including accuracy, computational efficiency, and industrial deployability.

#### 3.4.1. Accuracy Performance

In terms of accuracy-critical metrics, the proposed model achieved a mAP@0.5:0.95 of 0.952, outperforming APHS-YOLO by 0.6% and the original RT-DETR by 3.8%. The First Grade achieved a mAP@0.5:0.95 of 0.968,while the Second Group and Third Grade reached 0.951 and 0.937,respectively. Precision showed a similar trend, reaching 0.972—surpassing APHS-YOLO and the original RT-DETR by 0.1% and 4.3%, respectively. This superiority can be attributed to the model’s dual-module architecture: the LFFI module enhances the fidelity of large-scale mushroom mrophological features through wavelet-based feature integration, while the TSSA module preserves fine-grained details via statistical attention mechanisms.

**Figure 5:**
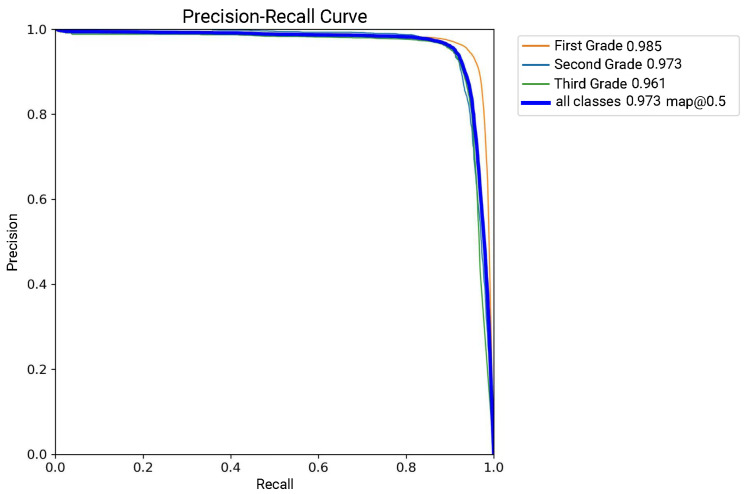
Precision–recall curve of Improved RT-DETR model.

**Figure 6:**
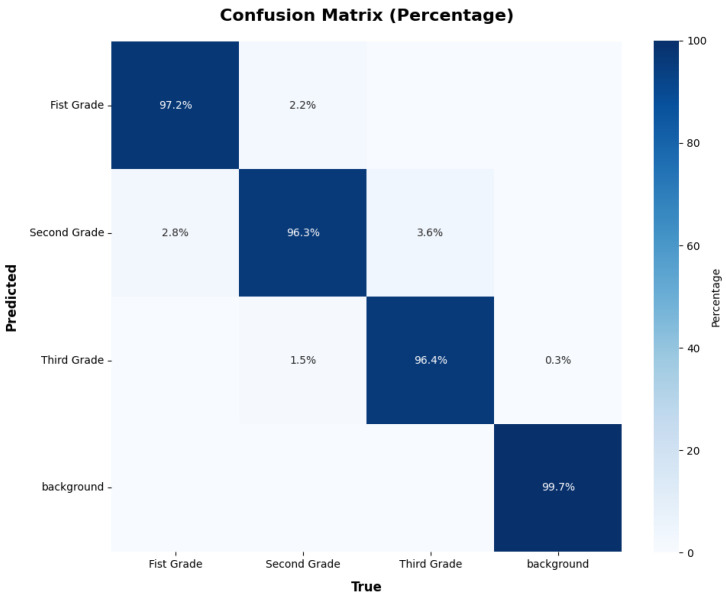
Normalized confusion matrix of Improved RT-DETR model.

To further evaluate the model’s ability to detect mushroom grades, Figure 5 shows the precision–recall (P-R) curves of the model for the different classes of groups. The P-R curve of all classes is 0.973 for mAP@0.5, but there is some variation in performance across grades. The model achieves higher accuracy for the First Grade. We selected a batch of *Stropharia rugoso-annulata* for analysis, and the confusion matrix of the recognition results is presented in Figure 6. As shown, the algorithm achieves high recognition accuracy, with consistently strong performance across all grading categories.

#### 3.4.2. Computational Efficiency

The proposed framework further distinguishes itself in computational efficiency. With a computational load of 42.8G FLOPs, it reduces the burden by 27.3% compared to the original RT-DETR while maintaining a 50.0% higher efficiency than APHS-YOLO. PMU is minimized to 1.1 GB—half that of the original RT-DETR and 8.3% lower than APHS-YOLO. In terms of real-time performance, the model achieves a throughput of 262 FPS, exceeding APHS-YOLO and the original RT-DETR by 13.9% and 20.7%, respectively.

#### 3.4.3. Industrial Deployability

A pivotal advantage for industrial scenarios lies in the elimination of NMS post-processing. Unlike APHS-YOLO, which introduces latency due to heuristic filtering in NMS, our end-to-end transformer architecture streamlines the inference pipeline. Coupled with its compact parameter size (18.2 M) and low PMU, this design facilitates seamless integration into resource-constrained edge devices—such as agricultural sorting lines—making it highly suitable for large-scale industrial applications.

### 3.5. Visualization of Detection Results

**Figure 7:**
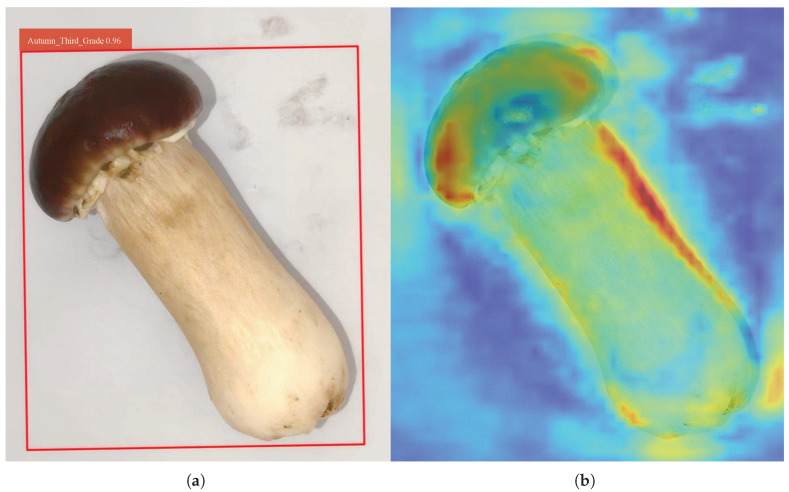
Visualization of detection results: (**a**) The detection result generated from the test result. (**b**) The attention heatmap generated from the test result.

We evaluated the model’s performance on the test set, and the detection result is shown in Figure 7a. As illustrated, the bounding box accurately delineates the target region, achieving an ideal detection outcome.

To provide an intuitive visualization of the model’s effectiveness, we generated an attention heatmap based on the test result (Figure 7b). The heatmap encodes feature response intensities using a color gradient, where warmer tones (e.g., red) indicate stronger attention to critical regions, and cooler tones (e.g., blue) indicate weaker responses. The heatmap was generated based on the activation intensity of the final detection layer.

As illustrated in Figure 7b, the Improved RT-DETR demonstrates superior attention consistency across both the cap and stalk regions of *Stropharia rugoso-annulata*, while effectively suppressing distractions from non-essential areas (e.g., background). This observation validates the effectiveness of our LFFI module in preserving low-frequency features for robust morphological representation. Notably, the model exhibits enhanced sensitivity to discriminative fine-grained features—particularly cap margins and stalk textures—which serve as critical grading criteria. These results demonstrate that our TSSA mechanism successfully maintains local feature discriminability while achieving computational efficiency.

The heatmap analysis substantiates that the synergistic integration of LFFI and TSSA addresses two key limitations of existing approaches: (1) the original RT-DETR’s inadequate global feature integration for large targets, and (2) APHS-YOLO’s compromised local detail preservation. Our solution achieves an optimal balance between holistic structure comprehension and fine-grained detail extraction—both essential for accurate mushroom grading.

## 4. Conclusions and Future Work

### 4.1. Conclusions

This study addresses the critical challenges in automated grading of *Stropharia rugoso-annulata*, by proposing an Improved RT-DETR model tailored to balance accuracy, efficiency, and edge deployability. Through targeted innovations and systematic validation, the key contributions are summarized as follows:

First, novel module design for large-target detection. The low-frequency feature integrator (LFFI) module is introduced to preserve low-frequency global structural information of large mushrooms, which is often lost in traditional CNN-based backbones. By leveraging wavelet decomposition to extract and integrate low-frequency components (LL sub-band) into convolutional feature streams, LFFI enhances the model’s ability to capture overall morphology—critical for accurate grading of large specimens. Ablation experiments (Table 3) confirm that LFFI alone improves mAP@0.5:0.95 by 3.0% (from 0.917 to 0.947), demonstrating its effectiveness in global structure capture.

Second, efficient attention mechanism for edge deployment. The Token Statistics Self-Attention (TSSA) module replaces traditional self-attention with second-moment statistics, reducing computational complexity from $O(n^{2})$ to O(n). This modification not only lowers FLOPs, but also naturally generates interpretable attention patterns.

Third, synergistic integration of modules for superior performance. The combined deployment of LFFI and TSSA yields a significant performance leap: the improved model achieves 95.2% mAP@0.5:0.95, outperforming the original RT-DETR and APHS-YOLO. Notably, it reduces computational overhead by 27.3% in FLOPs and 9.9% in parameters compared to the original RT-DETR, while maintaining 262 FPS—sufficient for real-time industrial sorting. This balance stems from LFFI’s global structural awareness complementing TSSA’s local detail sensitivity, resolving the long-standing trade-off between accuracy and efficiency in large-target agricultural detection.

Fourth, enhanced model interpretability and practicality. Unlike black-box models, TSSA’s attention mechanism, rooted in explicit objective optimization, leverages second-order moment statistics to achieve interpretable low-rank projection and soft clustering. This interpretability not only validates the model’s decision logic but also facilitates trust in real-world applications. Additionally, the elimination of NMS post-processing simplifies deployment, reducing latency and hardware requirements.

Collectively, these results demonstrate that the Improved RT-DETR offers a promising and scalable solution for automated grading of large-target crops such as *Stropharia rugoso-annulata*. While the findings highlight strong potential for practical deployment, we acknowledge that full industrial applicability requires further validation on diverse datasets and real production lines, which will be the focus of future work.

### 4.2. Future Work

While this study achieves significant advancements, several directions remain to further enhance the model’s robustness and applicability:

**Dataset expansion and algorithm verification.** In future work, it is necessary to further expand the existing dataset to reduce the risk of model overfitting. At the same time, the algorithm proposed in this study should be applied to public datasets for more extensive evaluation and verification of its generalization performance.

**Model interpretability and trustworthiness.** While this study provides qualitative visualization results to demonstrate feature enhancement, the interpretability analysis remains limited. In future work, we plan to incorporate quantitative interpretability tools such as Grad-CAM and SHAP to systematically evaluate the decision-making process of the proposed model. This will help validate whether the learned features align with grading criteria, thereby enhancing transparency, user trust, and practical adoption in industrial environments.

**Lightweight optimization for extreme edge deployment.** Despite reduced computational overhead, the improved model is still heavier than ultra-lightweight models like APHS-YOLO. Although our results demonstrate that TSSA reduces theoretical complexity from quadratic to linear, we acknowledge that practical deployment evidence remains limited. Specifically, the current study does not yet evaluate training time on resource-constrained GPUs or inference latency on edge devices, which are critical for industrial adoption. As part of our future work, we plan to benchmark TSSA across different hardware platforms, including low-memory GPUs and embedded devices such as Jetson Orin or Xavier NX, and to further optimize the module for real-time, on-device mushroom grading applications [34].

**Multi-modal fusion for fine-grained grading.** Grading accuracy could be enhanced by integrating complementary data modalities. For instance, fusing RGB images with depth information (via stereo cameras) would provide 3D morphological features (e.g., cap curvature, stalk thickness), while near-infrared (NIR) imaging could reveal internal quality indicators (e.g., moisture content). Integrating these modalities into the LFFI-TSSA framework—via cross-modal attention mechanisms—would enable more comprehensive grading criteria beyond visual morphology.

By advancing these directions, the proposed framework will not only solidify its position as a state-of-the-art solution for *Stropharia rugoso-annulata* grading but also contribute to the broader field of automated agricultural product processing, promoting efficiency and standardization in post-harvest workflows.

## Figures and Tables

**Table 1:** Dataset distribution of *Stropharia rugoso-annulata* grading.

Grade	Train	Validation	Test	Total	Ratio (%)
First Grade	2500	300	300	3100	33.8
Second Grade	2700	350	350	3400	37.1
Third Grade	2136	267	267	2670	29.1
**Total**	7336	917	917	9170	100

**Table 2:** Grading criteria of *Stropharia rugoso-annulata*. Grading is based on RDHP and RLDS ranges; RLDS prevails in conflicts.

Grade	RDHP	RLDS
First Grade	1.5∼2.5	0∼1.5
Second Grade	1.0∼1.5	1.5∼2.5
Third Grade	0∼1.0	>2.5

**Table 3:** Hyperparameter settings using in model training experiment.

Hyperparameter	Configuration
Optimizer	SGD
Batch Size	32
Epoch	150
Image Size	640 × 640
Learning Rate	0.01
Workers	8

**Table 4:** Ablation experiments for Improved RT-DETR.

RT-DETR	AIFI	TSSA	LFFI	Params (M)	FLOPs (G)	FPS	P	mAP (0.5:0.95)
**✓**	**✓**	**✗**	**✗**	20.2	58.9	217	0.932	0.917
**✓**	**✓**	**✗**	**✓**	20.5	61.3	208	0.961	0.947
**✓**	**✗**	**✓**	**✗**	17.8	40.1	267	0.949	0.924
**✓**	**✗**	**✓**	**✓**	18.2	42.8	262	0.972	0.952

**Table 5:** Comparison experiments for Improved RT-DETR.

Model	Params (M)	FLOPs (G)	FPS	PMU	P	mAP (0.5:0.95)
Improved RT-DETR	18.2	42.8	262	1.1	0.972	0.952
APSH-YOLO	5.5	21.4	230	1.2	0.963	0.944
RT-DETR	20.2	58.9	217	2.2	0.932	0.917

## Data Availability

The original contributions presented in the study are included in the article; further inquiries can be directed to the corresponding author.
